# A graph kernel method for DNA-binding site prediction

**DOI:** 10.1186/1752-0509-8-S4-S10

**Published:** 2014-12-08

**Authors:** Changhui Yan, Yingfeng Wang

**Affiliations:** 1 Department of Computer Science, North Dakota State University, Fargo, ND, 58108, USA; 2 Department of Computer Science, Utah State University, Logan, UT 84322, USA

**Keywords:** DNA-binding, kernel method, prediction

## Abstract

**Background:**

Protein-DNA interactions play important roles in many biological processes. Computational methods that can accurately predict DNA-binding sites on proteins will greatly expedite research on problems involving protein-DNA interactions.

**Results:**

This paper presents a method for predicting DNA-binding sites on protein structures. The method represents protein surface patches using labeled graphs and uses a graph kernel method to calculate the similarities between graphs. A new surface patch is predicted to be interface or non-interface patch based on its similarities to known DNA-binding patches and non-DNA-binding patches. The proposed method achieved high accuracy when tested on a representative set of 146 protein-DNA complexes using leave-one-out cross-validation. Then, the method was applied to identify DNA-binding sties on 13 unbound structures of DNA-binding proteins. In each of the unbound structure, the top 1 patch predicted by the proposed method precisely indicated the location of the DNA-binding site. Comparisons with other methods showed that the proposed method was competitive in predicting DNA-binding sites on unbound proteins.

**Conclusions:**

The proposed method uses graphs to encode the feature's distribution in the 3-dimensional (3D) space. Thus, compared with other vector-based methods, it has the advantage of taking into account the spatial distribution of features on the proteins. Using an efficient kernel method to compare graphs the proposed method also avoids the demanding computations required for 3D objects comparison. It provides a competitive method for predicting DNA-binding sites without requiring structure alignment.

## Background

Structural genomics projects are yielding an increasingly large number of protein structures with unknown function. As a result, computational methods for predicting functional sites on these structures are in urgent demand. There has been significant interest in developing computational methods for identifying amino acid residues that participate in protein-DNA interactions based on combinations of sequence, structure, evolutionary information, and chemical and physical properties. For example, Jones *et al*. [1] analyzed residue patches on the surface of DNA-binding proteins and used electrostatic potentials of residues to predict DNA-binding sites. Later, they extended that method by including DNA-binding structural motifs [2]. In related studies, Tsuchiya *et al*. [3] used a structure-based method to identify protein-DNA binding sites based on electrostatic potentials and surface shape. Gao and Skolnick [4] predict DNA-binding using structural template comparison and statistical potential. Sophisticated machine-learning methods, like SVM, neural network, and Random Forest, have also been used to predict DNA-binding sites integrating a wide range of features [5-9]. On another direction, several methods have been developed for predicting DNA-binding sites using only protein sequence-derived information as input [10-15]. To date, the methods that take the advantage of structure-derived information achieve better results than those using only sequence-derived information.

One common limitation of the above-mentioned methods is that the sequence and structural properties of a surface patch are input to machine-learning methods in the form of vectors. When the properties of a surface patch are encoded as a vector, the information of how these properties distribute over the surface is lost. For example, if a surface patch includes five amino acid residues, the above-mentioned methods will encode the amino acid identities of this surface patch as five independent values in a vector. In this representation, the spatial arrangement of these five residues on the surface patch is not encoded. Unfortunately, the spatial arrangement of properties on a surface patch plays a crucial role in determining the function of the surface patch.

To overcome this limitation, this paper presents a graph approach for DNA-binding site prediction. In this study, graphs are used to represent surface patches, such that the spatial arrangement of various properties on the surface is explicitly encoded. The similarities between surface patches are then computed using a graph kernel method. A voting strategy is then used to classify surface patches into DNA-binding sites versus non-binding sites based on their similarity to known DNA-binding surface and non-DNA-binding surface. When applied to set of unbound structures of DNA-binding proteins, the proposed method can precisely identify the locations of DNA-binding sites.

## Methods

### DNA-binding proteins

DNA-binding proteins were obtained from our previous study [10]. In that study, we extracted all protein-DNA complexes from the PDB [16]. Then, the dataset was culled using PISCES [17]. The resulting dataset consisted of 171 proteins with mutual sequence identity ≤ 30% and each protein had at least 40 amino acid residues. All the structures have resolution better than 3.0 Å and R factor less than 0.3. In the current study, seven features are evaluated for their usefulness in the prediction of DNA-binding sites. Thus, seven features were calculated for each protein. Among them, structural conservation was calculated based on the alignment of structural neighbors (See details in section 2.2). 25 proteins were discarded because no structures neighbors were found. In the end, 146 DNA-binding proteins were used to evaluate the proposed method in cross-validation.

### Features

DNA was removed from the protein-DNA complexes and seven features were calculated for each amino acid of the protein: (1) Relative solvent accessibility was calculated using NACCESS [18]; (2) Electrostatic potential was calculated using Delphi [19] with the same parameters used in the study of Jones et a. [1]. The electrostatic potential of a residue is defined as the average of the electrostatic potentials at the locations of all its atoms as described in Jones et a. [1]; (3) Sequence entropy at each residue position (the sequence entropy for the corresponding column in the multiple sequence alignment) was extracted from the HSSP database [20]. Sequence entropy is a measure of sequence conservation. The lower the value, the more conserved is the corresponding residue position; (4) Surface curvature at each residue position was calculated using MSP (http://connolly.best.vwh.net/); (5) Pockets on protein surface were detected using Proshape (http://csb.stanford.edu/~koehl/ProShape/download.php). The pocket size of a residue is defined as the size of the pocket that the residue is located in. If a residue is not located in any pocket, then a value of 0 is assigned to the pocket size of the residue; (6) The DALI server [21] was used to search for structural neighbors in the PDB for each of the DNA-binding proteins. The DALI server returned a multiple alignment of the query structure and its structural neighbors. Then, structural conservation score was calculated for each residue position using Scorecons [22] based on the multiple alignment; and (7) position-specific scoring matrix (PSSM) of a protein was built by running 4 iterations of PSI-BLAST [23] against the NCBI non-redundant (nr) database. In the PSSM, each residue position corresponds to 20 values. Thus, in total, each amino acid residue is associated with 26 attributes. All these attributes were normalized to the range of 0[1].

### Interface residues and surface residues

Interface residues are defined as in Jones *et al*. [1]. Solvent accessible surface area (ASA) was computed for each residue in the unbound protein (in absence of DNA) and in the protein-DNA complex. A residue is defined to be an interface residue if its ASA in the protein-DNA complex is less than its ASA in the unbound protein by at least 1Å^2^. A residue is defined to be a surface residue if its relative accessibility in the unbound protein is >5%. In total, 4,337 interfaces residues and 27,248 surface residues were obtained.

### Interface patches and non-interface patches

For each DNA-binding protein, an interface patch and a non-interface patch were obtained. The interface patch included all the interface residues. The non-interface patch was randomly taken from the protein surface such that (1) it consisted of a group of contiguous surface residues; (2) it had the same number of residues as the interface patch; and (3) it did not include any interface residue.

### Graph representation of patches

Each amino acid residue is represented using a node labeled with the 26 attributes of the residue. Two residues are considered contacting if the closest distance between their heavy atoms is less than the sum of the radii of the atoms plus 0.5 Å. An edge is added between two nodes if the corresponding residues are contacting. In this way, a surface patch of residues is represented as a labeled graph.

### Graph kernel

Kernel methods are a popular method with broad applications in data mining. In a simple way, a kernel function can be considered as a positive definite matrix that measures the similarities between each pair of input data. It the currently study, a graph kernel method, namely shortest-path kernel, developed by Borgwart and Kriegel [24], is used to compute the similarities between graphs.

The first step of the shortest-path kernel is to transform original graphs into shortest-path graphs. A shortest-path graph has the same nodes as its original graph, and between each pair of nodes, there is an edge labeled with the shortest distance between the two nodes in the original graph. In the current study, the edge label will be referred to as the weight of the edge. This transformation can be done using any algorithm that solves the all-pairs-shortest-paths problem. In the current study, the Floyd-Warshall algorithm was used.

Let G_1 _and G_2 _be two original graphs. They are transformed into shortest-path graphs S_1_(V_1_, E_1_) and S_2_(V_2_, E_2_), where V_1 _and V_2 _are the sets of nodes in S_1 _and S_2_, and E_1 _and E_2 _are the sets of edges in S_1 _and S_2_. Then a kernel function is used to calculate the similarity between G1 and G2 by comparing all pairs of edges between S_1 _and S_2_.

$$K\left(G_{1},G_{2}\right)=\underset{e_{1}\in E_{1}}{\sum }\underset{e_{2}\in E_{2}}{\sum }k_{edge}\left(e_{1},e_{2}\right)$$

where, *k_edge_*( ) is a kernel function for comparing two edges (including the node labels and the edge weight).

Let e_1 _be the edge between nodes v_1 _and w_1_, and e_2 _be the edge between nodes v_2 _and w_2_. Then,

$$k_{edge}\left(e_{1},e_{2}\right)=k_{node}\left(v_{1},v_{2}\right)*k_{weight}\left(e_{1},e_{2}\right)*k_{node}\left(w_{1},w_{2}\right)$$

where, *k_node_*( ) is a kernel function for comparing the labels of two nodes, and *k_weight_*( ) is a kernel function for comparing the weights of two edges. These two functions are defined as in Borgward et al.[25]:

$$k_{node}\left(v,w\right)=\text{exp}\left(-\frac{||labels\left(v\right)-labels\left(w\right)|{|}^{2}}{2\delta^{2}}\right)$$

where, *labels*(*v*) returns the vector of attributes associated with node *v*. When *n *features were used to labeled the nodes, *labels*(*v*) and *labels(w) *could be considered as the coordinates of two points in the *n*-dimensional space, and ||*labels(v)-labels(w)*|| is the Euclidean distance between the two points. Note that *K_node_*() is a Gaussian kernel function. We tried different values for $\frac{1}{2\delta^{2}}$ between 32 and 128 with increments of 2, the accuracy varied from 86% to 88.7% when all features were used in the cross validation. When $\frac{1}{2\delta^{2}}$ was set to 72 the best accuracy was achieved.

$$k_{weight}\left(e_{1},e_{2}\right)=\text{max}\left(0,c-|weight\left(e_{1}\right)-weight\left(e_{2}\right)|\right)$$

where, *weight*(*e*) returns the weight of edge *e. K_weight_*( ) is a Brownian bridge kernel that assigns the highest value to the edges that are identical in length. Constant *c *was set to 2 as in Borgward et al.[25]. We tried different values of *c *between 1 and 5 with increments of 1, the change in accuracy was less than 1%.

### Classification

When the shortest-path graph kernel is used to compute similarities between graphs, the results are affected by the sizes of the graphs. Consider the case that graph G is compared with graphs G_x _and G_y _separately using the graph kernel:

$$K\left(G,G_{x}\right)=\underset{e\in E}{\sum }\underset{e_{x}\in E_{x}}{\sum }k_{edge}\left(e,e_{x}\right)$$

$$K\left(G,G_{y}\right)=\underset{e\in E_{}}{\sum }\underset{e_{y}\in E_{y}}{\sum }k_{edge}\left(e,e_{y}\right)$$

If G_x _has more nodes than G_y _does, then |E_x_|>|E_y_|, where E_x _and E_y _are the sets of edges in the shortest-path graphs of G_x _and G_y_. Therefore, the summation in *K(G, G_x_) *includes more items than the summation in *K(G, G_y_) *does. Each item (i.e., *k_edge_( )*) inside the summation has a non-negative value. The consequence is that if *K(G, G_x_)>K(G,G_y_) *it may not necessary indicate that G*_x _*is more similar to G than G*_y _*is, instead, it could be an artifact of the fact that G_x _has more nodes than G_y_. To overcome this problem, a voting strategy is developed for predicting whether a graph (or a patch) is an interface patch:

**Algorithm **Voting_Stategy (G)

**Input**: graph G

**Outpu**t: G is an interface patch or non-interface patch

Let T be the set of proteins in the training set

Let v be the number of votes given to "G is an interface patch"

v = 0

While (T is not empty)

{

Take one protein (P) out of T

*Let G_int _and G_non-int _be the interface and non-interface patches from P*.

If K(G, G_int_)>K(G,G_non-int_), then increase v by 1

}

If v>|T|/2, then G is an interface patch

Else G is a non-interface patch

Using this strategy, when *K(G, G_int_) *is compared with *K(G, G_non-int_)*, G_int _and G_non-int _are guaranteed to have identical number of nodes, since they are the interface and non-interface patches extracted from the same protein (see section 2.4 for details). Each time *K(G, G_int_)>K(G, G_non-int_) *is true, one vote is given to "G is an interface patch". In the end G is predicted to be an interface patch if "G is an interface patch" gets more than half of the total votes, i.e., v>|T|/2.

## Results and discussion

### Distinguish interface patches from non-interface patches

146 interface patches and 146 non-interface patches were obtained from the dataset. The graph kernel method was used to compute similarities between patches and the voting strategy was used to classify these patches into interface versus non-interface patches. When evaluated using a leave-one-out cross-validation, this method achieves an overall accuracy of 88.7%. 87.7% (Sensitivity) of the interface patches and 89.7% (Specificity) of the non-interface patches were correctly predicted.

### Contributions of the features

In the above experiment, all seven features were used to calculate similarities between graphs. To evaluate the importance of each feature, the leave-one-out cross-validation was repeated with only one feature being used at one time. Table 1 shows show that when only one feature is used, the method achieves the best performance (86.9% accuracy) with PSSM as input. When all seven features are used, the method achieves the highest accuracy (88.7%).

**Table 1:** Contributions of features.

Features	**PSSM**^1^	E_P^2^	Ent^3^	StrCn^4^	rASA^5^	Cur^6^	Poc^7^	All^8^
Accuracy (%)	86.9	77.0	67.5	54.7	54.5	54.1	54.1	88.7

^1 ^PSSM: position-specific scoring matrix; ^2 ^E_P^: ^electrostatic potential; ^3 ^Ent: sequence entropy; ^4 ^StrCn: structural conservation; ^5 ^rASA: relative solvent accessibility; ^6 ^Cur: surface curvature; ^7 ^Poc: size of the pocket where the residue is located; and ^8 ^All: all the seven attributes were used.

### Prediction of DNA-binding residues

**Figure 1:**
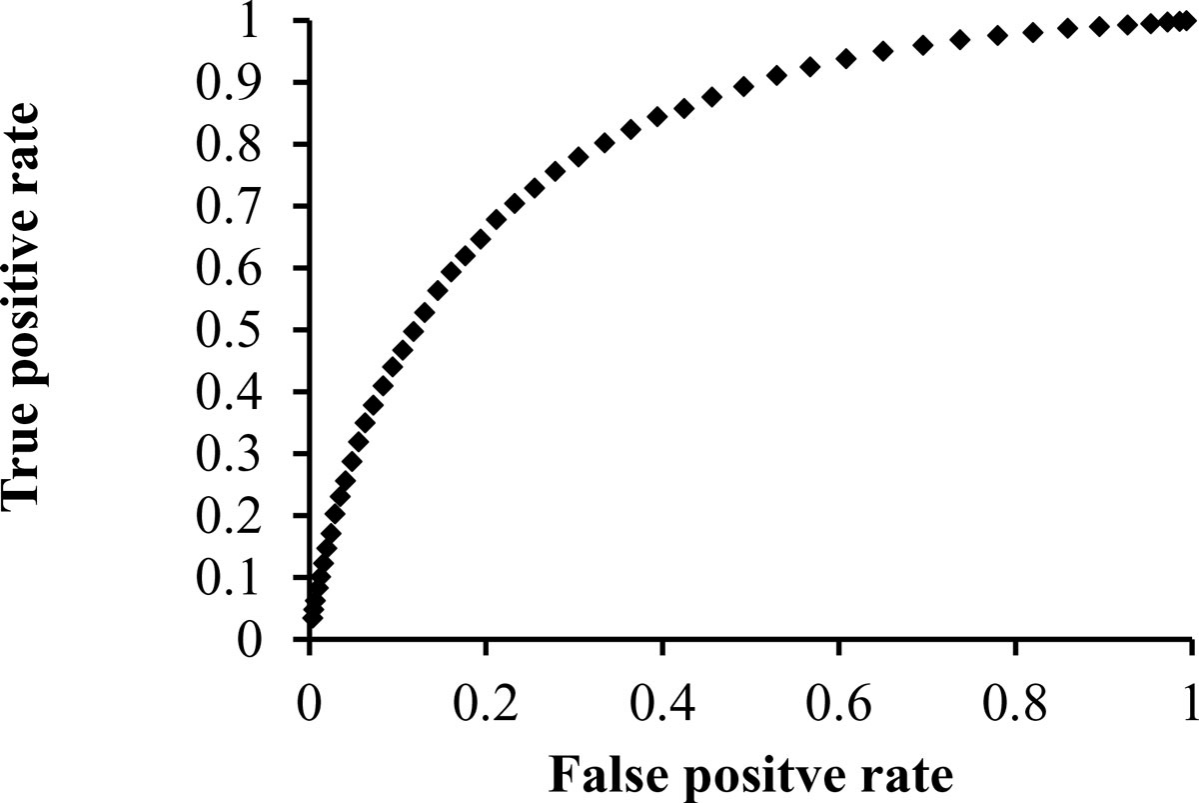
**The ROC of the proposed method in predicting DNA-binding site residues**.

The interface and no-interface patches used in aforementioned experiments were generated based on actual binding sites. In a practical prediction situation, that size and the shape of the binding site are unknown. In order to evaluate the proposed method's ability to discover DNA-binding sites in proteins, for each surface residue, we generated a surface patch that included the residue and its nearest 5 neighbors. Then, the 146 interface and 146 non-interface patches were used as the training set to classify the surface patches into DNA-binding and non-DNA-binding classes. Leave-one-out cross validation was performed at the protein level, so that interface and non-interface patches from a protein were not used in the classification of surface patches from the same protein. The proposed method identified DNA-binding residues with 79.5% accuracy, 84.9% specificity and 51.5% sensitivity. By changing the classification threshold (i.e. v>threshold) using in voting strategy, we obtained the ROC for the prediction (Figure 1). The AUC of the ROC is 0.80.

### Predicting DNA-binding sites on unbound proteins

13 test proteins with both DNA-bound and unbound structures in the PDB were taken from a previous study [7]. 14 such proteins were considered in the study by Tjong and Zhou. Here, we discarded 2abk because the sequence identity between the bound and unbound proteins was only 45%. In this section, the DNA-binding sites on the 13 unbound proteins will be predicted using the graph kernel method. The prediction results are evaluated based on the actual DNA-binding sites gleaned from the corresponding protein-DNA complexes. For each surface residue on the test proteins, we obtained a surface patch that included the residue and its 5 closest neighbors. Then, the patches were classified into interface versus non-interface patches using the 146 proteins as training set. For each test protein, the training set was filtered such that none of the proteins in the training set shares > 30% identical residues with the test proteins.

For 8 of the 13 proteins (*Italic *in Table 2), DALI was not able to find structural neighbors in PDB. Thus, the structural conservation of these proteins could not be computed. For these 8 proteins, only PSSM was used to compute similarities in the graph kernel, since table 1 shows that the proposed method can still achieve high accuracy when only PSSM is used in the graph kernel. For the remaining 5 proteins, all seven features were used in the graph kernel.

**Table 2:** Predictions by the top 1 patch.

Unbound PDB id ^1^	Bound PDB id	Top 1 patch		*P_Random _*(%) ^4^
		**TP^2^**		

1iknA,C	1leiA,B	6	0	0.4

*1g6nA,B*	1zrfA,B	6	0	2.4

1zzkA	1zziA	6	0	2.9

*1mml*	1ztwA	4	2	4.2

1a2pC	1brnL	6	0	4.5

*1ko9A*	1m3qA	6	0	4.9

*1qc9A*	1cl8A,B	4	2	8.0

1lqc	1l1mA,B	6	0	8.7

*1qzqA*	1rfiB	2	4	9.5

*1xx8A*	1xyiA	6	0	10.7

1qqiA	1gxpA,B	4	2	19.3

*1l3kA*	1u1qA	3	3	25.6

*2alcA*	1f5eP	4	2	26.7

^1 ^For the proteins in *italic*, the interfaces were predicted using only PSSM. For the others, all seven features were used; ^2 ^TP: the number of the interface residues falling in the top 1 patch; ^3 ^FP: the number of non-interface residues in the top 1 patch. ^4 ^*P_random_*: When a patch is randomly picked, the probability of it containing at least as many interface residues as the top 1 patch.

#### The top 1 patch overlaps with the actual DNA-binding site

Using the voting strategy, each patch was assigned a number representing the number of votes it got. The higher the vote number, the more similar is the patch to the interface patches. For each test protein, we sorted the patches based on the numbers of votes they get, such that the top 1 patch got the most votes. Table 2 shows that on every test protein, the top 1 patch overlaps with the actual DNA-binding site. On 7 of the 13 proteins, all the six residues in the top 1 are actually interface resides (6 true positives, 0 false positive). When averaged over the 13 proteins, the top 1 patch contains 4.8 interface residues and 1.2 non-interface residues, i.e., on average, 80% of the residues in the top 1 patch are interface residues. These results show that on a test protein, the top 1 patch can precisely indicate the location of the actual DNA-binding site.

If a patch is randomly picked from a test protein, what is the probability (*P_random_*) to obtain a patch that is at least as good as the top 1 patch in terms of predicting the DNA-binding sites? For each test protein, *P_random _*is calculated as *N/N_all _*, where *N_all _*is the total number of patches on the protein, *N *is the number of the patches that have at least as many interface residues as the top 1 patch. The results (Table 2) show that for 9 of the 13 proteins, *P_random _*is less than 10%. The average *P_random _*for the 13 protein is 9.8%. This indicates the significance of the predicting method.

#### Obtaining higher coverage by combining multiple top-ranking patches

In the evaluation of DNA-binding site prediction methods, there are mainly two measures that researchers are interested in: *coverage (TP/N_int_) *and *accuracy (TP/N_pr_)*, where *TP *is true positive, i.e. the number of residues that are predicted to be interface residues and are actually interface residues, *N_int _*is the total number of interface residues and *N_pr _*is the number of residues that are predicted to be interface residues. Coverage shows percentage of the actual interface residues that are correctly predicted and accuracy is the percentage of the predicted interface residues that are actually interface residues.

**Figure 2:**
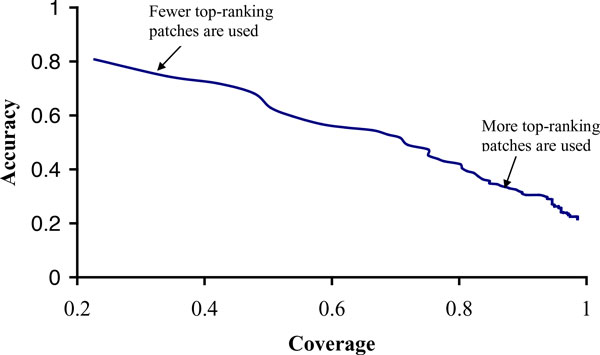
**Tradeoff between coverage and accuracy for the proposed method**.

The above section has shown that the top 1 patch can precisely indicate the location of the DNA-binding site on each test protein. However, since a patch has only 6 residues, the predictions solely based on the top 1 patch only have low coverage. We can obtain higher coverage by combining the predictions of multiple top-ranking patches. For example, if only top 1 patch is used to predict DNA-binding sites, the average coverage and accuracy are 23% and 81% for the 13 proteins. When the union of the top 3 patches is used to predict DNA-binding sites, coverage increases to 42%, but accuracy decreases to 72%. Figure 2 shows the tradeoff between coverage and accuracy when multiple top-ranking patches are used. Figure 2 shows the trend that as more top-ranking patches are used, coverage increases but accuracy decreases. If researchers prefer to identify more interface residues at the cost of lower accuracy, then they can choose to use more top-ranking patches to predict DNA-binding sites. The performance will fall at the right side of the curve. On the other hand, if they desire higher accuracy, then they can use fewer patches.

#### Comparison with other methods

While many computational methods have been proposed for the prediction of DNA-binding sites, it is difficult to make direct comparisons between them, due to lack of a standardized benchmark for the evaluation. Here, it is not our intent to make a systematic comparison between different methods. We only compared our method with two recent methods, MV [9] and DISPLAR [7], regarding their ability to find DNA-binding sites on the 13 unbound proteins. Both MV and DISPLAR use both structural and sequence information in predicting DNA-binding sites.

The MV method [9] integrates a wide range of structural, evolutionary, energy-based and experimental data and uses a random forest method to predict functional sites, including protein-, peptide-, DNA-, RNA-binding sites on protein structures. The 13 unbound protein structures were submitted to the MV online server to obtain the predicted DNA-binding sites. The MV returned a list of amino acid residues with their corresponding prediction scores. By changing the score threshold using for prediction, the MV method obtained an AUC of 0.85 for the ROC. In comparison, our method returned a list of surface patches with their prediction scores (i.e. vote counts). Our method achieved a slightly better AUC of 0.87.

The 13 test proteins used in this study were also used to evaluate the DISPLAR method when Tjong and Zhou proposed it [7]. In their publication, Tjong and Zhou also used coverage and accuracy to evaluate the predictions. However, they defined accuracy using a loosened criterion of "true positive" such that if a predicted interface residue is within four nearest neighbors of an actual interface residue, then it is counted as a true positive. Here, in the comparison of the two methods, the strict definition of true positive is used, i.e., a predicted interface residue is counted as true positive only when it is an actual interface residue. The original data were obtained from table 1 of Tjong and Zhou [7], the accuracy for the neural network method was recalculated using this strict definition (Table 3).

**Table 3:** Comparison with other methods.

Proteins	Graph Kernel^1^		DISPLAR^2^		MV^3^	
	**Coverage**	**Accuracy**	**Coverage**	**Accuracy**	**Coverage**	**Accuracy**

1a2p	**0.45**^4^	**0.90**	0.44	0.33	0.45	0.45

1ikn	**0.46**	**0.60**	0.46	0.38	0.46	0.39

1lqc	0.95	0.56	**0.95**	**0.68**	0.95	0.49

1qqi	0.70	0.67	**0.75**	**0.66**	0.70	0.33

1zzk	**0.40**	**0.53**	0.37	0.47	**0.40**	**0.53**

1qc9	0.55	0.41	**0.58**	**0.66**	0.55	0.23

2alc	**0.90**	**0.67**	0.90	0.59	0.75	0.47

1ko9	**0.50**	**1.00**	0.48	0.80	0.50	0.89

1qzq	0.57	0.63	0.57	0.24	**0.57**	**1.00**

1l3k	0.44	0.56	**0.44**	**0.68**	0.44	0.63

1xx8	**0.74**	**0.93**	0.60	0.92	0.74	0.32

1g6n	0.48	0.73	**0.77**	**0.75**	0.48	0.52

*1 mml*	*0.46*	*0.35*	*0.38*	*0.50*	*0.46*	*0.40*

^1 ^The method proposed in this study; ^2 ^The DISPLAR method developed by Tjong and Zhou [7]; ^3 ^The MV method developed by [9]; ^4 ^The bold font shows the best performance among the three methods on each protein.

MV and our method returned a list of residues (or patches) with decreasing prediction scores and allowed users to tradeoff between coverage and accuracy by choosing a threshold. To compare them with DISPLAR, for each test protein, we gradually decreased the prediction threshold until the coverage achieved was equal to or higher than that of DISPLAR. Then the coverage and accuracy of the methods were compared. On a test protein, method A is better than B, if *accuracy(A)>accuracy(B) *and *coverage (A)≥coverage(B)*. Table 3 shows the comparisons. On each protein, the best performance among the three methods are shown in the bold font. On 1 mml no method is better than others are on both accuracy and coverage, thus a best performance cannot be identified. Our method achieved the best performance in 6 of the 13 proteins (tie with MV on 1zzk).

## Conclusions

This paper presents competitive method for predicting DNA-binding sites on proteins. The effectiveness of the method is demonstrated using cross-validation and by applying it to 13 unbound protein structures. Different from other methods that represent sequence and structural properties of surface using vectors, the method proposed in this study uses labeled graphs. Compared to vectors, one advantage of labeled graphs is that they can specifically encode the spatial arrangement of the properties on protein surface. Since proteins and DNA interact in a 3-dimensional space, the spatial arrangement of the properties on protein surface plays a pivotal role in the interactions. Therefore, computational methods for prediction of the interface should consider the spatial arrangement of the properties. The proposed method uses a graph kernel to explore this information. Using this graph kernel method, the proposed method avoids the demanding computation involved in the structural alignment and comparison.

## Competing interests

The authors declare that they have no competing interests.

## Authors' contributions

CY conceived of and designed the study, performed the computation and analysis, and drafted the manuscript. YW made partially contribution to the programming. All authors read and approved the final manuscript.

## Declarations

Publication costs for this work were funded by NSF award 1262265 to C. Yan.

This article has been published as part of *BMC Systems Biology *Volume 8 Supplement 4, 2014: Thirteenth International Conference on Bioinformatics (InCoB2014): Systems Biology. The full contents of the supplement are available online at http://www.biomedcentral.com/bmcsystbiol/supplements/8/S4.
